# Two new remarkable genera of Oriental Pentastirini (Hemiptera, Auchenorrhyncha, Cixiidae)

**DOI:** 10.3897/zookeys.1292.206598

**Published:** 2026-09-21

**Authors:** Bence Péter Schlitt, András Orosz

**Affiliations:** 1 Institute of Biology, Eötvös Loránd University, Pázmány Péter sétány 1/A, H-1117, Budapest, Hungary Institute of Biology, Eötvös Loránd University Budapest Hungary; 2 Hemiptera Collection, Department of Zoology, Hungarian National Museum Public Collection Centre – Hungarian Natural History Museum, Baross u. 13, H-1088 Budapest, Hungary Hemiptera Collection, Department of Zoology, Hungarian National Museum Public Collection Centre – Hungarian Natural History Museum Budapest Hungary

**Keywords:** Delphacoidea, Fulgoromorpha, morphology, new genus, new species, Pentastirinae, planthoppers, taxonomy.

## Abstract

In this paper, we describe two new genera and three new species in the planthopper tribe Pentastirini Emeljanov, 1971 (Hemiptera: Cixiidae: Pentastirinae) from Southeast Asia. The genus *Pteroprocessus***gen. nov**. is established for *Pteroprocessus nabhitabhatai***gen. et sp. nov**. from Trang Province, Thailand, along with the description of *Pteroprocessus obscurinervis***gen. et sp. nov**. from Yunnan Province, China. *Indolipa binghami* (Distant, 1911) is transferred to the newly established genus based on external morphological homologies and the new combination *Pteroprocessus binghami* (Distant, 1911) **gen. et comb. nov**. is proposed. In addition, a new genus, *Philippinarus***gen. nov**. is proposed for *Philippinarus granulatus* (Muir, 1924) **gen. et comb. nov**., and a second species, *Philippinarus tarandiformis***gen. et sp. nov**. is described from the Philippines.

## Introduction

The subfamily Pentastirinae Emeljanov, 1971 was recently elevated to subfamily rank by Emeljanov and Shcherbakov (2025) and currently includes two tribes, Pentastirini Emeljanov, 1971 and Mnemosynini Emeljanov, 1992. Recent phylogenetic investigations of the family Cixiidae Spinola, 1839 published by Luo et al. (2024) revealed a more detailed understanding of relationships among cixiid lineages, showing that Pentastirini forms the so-called “Pentastirinian lineage”, which is more closely related to the “Oecleinian lineage” (including Oecleini and Bothriocerini) than to Mnemosynini, the latter forming a clade with Borysthenidae Emeljanov, 1989 referred to as the “Borysthenian lineage”. These results suggest that the current classification of Cixiidae requires further reconsideration. As phylogenetic-based taxonomic changes have not yet been formally established, we follow here the classification proposed by Emeljanov and Shcherbakov (2025).

The tribe Pentastirini Emeljanov, 1971 currently comprises 58 extant genera and 753 extant species worldwide (Dmitriev et al. 2026). The Oriental Pentastirini have been studied by numerous authors, including Matsumura (1914), Muir (1922, 1924, 1925), Jacobi (1941, 1944), Fennah (1978), and Van Stalle (1989, 1991). The first comprehensive revision of the Oriental fauna was provided by Van Stalle (1991), whose meticulously detailed monograph substantially expanded the knowledge of the group. Nevertheless, the generic placement of numerous species remains uncertain, as several genera are still considered paraphyletic and many taxa were historically assigned to the catch-all genus *Oliarus* Stål, 1862 (Hoch 2006; Löcker et al. 2006).

In an effort to clarify the taxonomic framework of *Oliarussensu lato*, Emeljanov (2001) established several new genera, followed by further contributions from Hoch (2006) and Emeljanov (2007). In addition, Löcker et al. (2006) revised the Australasian Pentastirini and provided important comparative data for Oriental taxa. Despite these significant advances, the actual diversity of Oriental Pentastirini remains largely unknown.

In the present study, we describe two remarkable new genera of Pentastirini from Southeast Asia. The new genus *Pteroprocessus* gen. nov. is distinguished by a unique combination of morphological characters unknown in other genera of the subfamily and includes three species (two of them newly described in this paper): *Pteroprocessus binghami* (Distant, 1911) gen. et comb. nov., *P. nabhitabhatai* gen. et sp. nov. and *P. obscurinervis* gen. et sp. nov. Furthermore, we establish *Philippinarus* gen. nov. for *Philippinarus granulatus* (Muir, 1924) gen. et comb. nov. and describe an additional species, *Philippinarus tarandiformis* gen. et sp. nov., from the Philippines.

## Material and methods

Morphological terminology for head, body and thorax follows Hendrix and Bartlett (2025) and Van Stalle (1991), for male genitalia Van Stalle (1991), and for female genitalia Bourgoin (1993). The wing venation terminology follows Bourgoin et al. (2014) and Le Cesne et al. (2022). Venation abbreviations are as follows: CA costa anterior; ScP + R subcosta posterior + radius; MP media posterior; CuA cubitus anterior; CuP cubitus posterior; Pcu postcubitus; A_1_ first anal vein; A_2_ second anal vein; pt pterostigma. Longitudinal veins are noted with capital letters, branches numbered adequately (e.g. CuA_1_ is the first branch of CuA), while veinlets (= crossveins) are written in lowercase italics (e.g. *rp-mp* is the veinlet between RP and MP).

The authors examined and identified the specimens using a Leica MZ6 stereomicroscope (Leica Microsystems, Wetzlar, Germany). All taxa are listed in alphabetical order. Detailed external morphological characters are given in the generic description of the genus, while the specific descriptions contain only the key differences.

Card-mounted dry specimens were used for the descriptions and illustrations. The abdomen of the examined specimens was removed and macerated in 10% KOH solution, rinsed successively in 96% ethanol and distilled water, and preserved in plastic microvials filled with glycerol. Body measurements are from the apex of the vertex to the tip of the forewing. Measurements are in millimetre (mm) and micrometre (μm). Photographs of specimens were taken using a Raynox Super Macro Conversion Lens DCR-250 adapter set (Yoshida Industry Co., Tokyo, Japan) attached to a Nikon D7200 digital camera (Nikon Corporation, Tokyo, Japan) managed with Helicon Remote, while photographs of wings, legs and male terminalia were taken with an Olympus SZX12 photographic microscope (Olympus Co., Tokyo, Japan), using the imaging software DPController and DPManager (Olympus Co., Tokyo, Japan). Photographs were stacked using Helicon Focus 8 (Helicon Soft, Kharkiv, Ukraine) and Adobe Photoshop. Figures were edited with GIMP v. 2.10.38 GNU Image Manipulation Program. Drawings were done under a Leica MZ6 stereomicroscope by the second author. The provided distributional map for new genera and species was made with QGIS Geographic Information System (v. 3.34.11) Open Source Geospatial Foundation (available from: https://qgis.org). The base map used in this study was the “MapTiler Satellite Plain” (available from: https://www.maptiler.com/maps/satellite/) layer, accessed via QGIS.

Specimen labels are given verbatim with explanations and additions in square brackets. Label information is quoted, with slashes (/) indicating line breaks and double slashes (//) indicating separate labels. Type labels are handwritten. Holotypes and all paratypes of the newly described species are provided with a red-framed label on white paper with printed red text “HOLOTYPUS” and “PARATYPUS”, respectively.

The specimens examined in this study were deposited in the following collections: Bernice P. Bishop Museum, Honolulu, Hawaii, USA (**BPBM**); Hungarian Natural History Museum, Budapest, Hungary (**HNHM**); Moravian Museum (Moravske Museum), Brno, Czech Republic (**MMBC**); Natural History Museum of the National Science Museum, Technopolis Khlong 5, Thailand (**THNHM**).

The institutional abbreviations were used (where possible) as listed in the Insects and Spider Collections of the World Website (Evenhuis 2026, accessed 19^th^ March 2026).

## Taxonomy

**Family Cixiidae Spinola, 1839**

**Subfamily Pentastirinae Emeljanov, 1971**

**Tribe Pentastirini Emeljanov, 1971**

### Genus Philippinarus gen. nov.

Taxon classificationAnimaliaHemipteraCixiidae

77FF8430-F7A1-5E41-9442-2A11A87C371E

https://zoobank.org/71326A82-C6AB-47BC-ADE0-C35361A1D672

#### Type species.

*Oliarus granulatus* Muir, 1924, designated here.

#### Description.

Medium-sized planthoppers (5.0–6.3 mm). Head slightly narrower than pronotum. Vertex longer than wide; margins elevated; anterior margin convex, posterior margin deeply concave. Median carina on vertex distinct, covering 2/3 of vertex. Eyes large, wider than long; medially wider than vertex; lateral ocelli distinct, large. Dorsolateral foveae roughly quadrate, posteriorly elongate, tapered; dorsoapical fovea conspicuously small, oval. Frons narrower above eyes, enlarged to postclypeus, widest under eyes; lateral margins elevated. Frons with distinct, thick median carina. Median carina on frons reaching upper third of postclypeus; forked below apical transverse carina, defining a distinct, subtriangular frontal fovea. Fenestrae indistinct; median ocellus well-developed. Epistomal suture convex. Postclypeus narrowing towards anteclypeus; with distinct horizontal striation; margins slightly elevated. Anteclypeus tapered. Frons and clypeus covered with fine setae. Rostrum projects beyond middle coxae. Pedicel large, globular; antennal flagellum thin, moderate in size. Pronotum thin, posterior margin deeply concave; margins distinctly elevated; widened behind eyes. Median carina of pronotum distinct, thick. Paranotal lobes large, subtriangular, apically tapered; margins slightly elevated. Mesonotum large, almost as long as wide, with five carinae; lateral carinae slightly convex, intermediate carinae incomplete; median carina straight, not reaching tip of scutellum. Scutellum indistinct, depressed. Mesonotum flattened in lateral view. Tegulae large, kidney-shaped. Macropterous. Forewings extend beyond abdomen. Pterostigma large, rounded, distinct. Veins of forewings with distinct granules along veins, each bearing small setae. Forewings with long ScP + R, forking into ScP + RA and RP around the first-level forking of CuA (or distinctly before); RA with three terminals; RP stem long, with two terminals; *ir* veinlet present between RA_2_ and last terminal of RP, almost as long as *rp-mp*, *rp-mp’* slightly longer; *rp-mp* present between forking of MP and midpoint of RP stem; *rp-mp’* present slightly basad of RP fork and last terminal of MP_1_. M stem long, forking well distad of ScP + RA and CuA forks, slightly basad of apex of clavus. M with two main branches forking into five terminals, M_1_ forking into two branches; two *im* veinlets present, equal in length. Stem of CuA almost as long as stem of ScP + R, slightly distad of midpoint of MP. CuA_1_ and CuA_2_ unforked; *icua* and *icu* present, subequal in length. Pcu and A_1_ fused basad of midpoint of clavus; Pcu + A_1_ longer than A_1_, reaching anal margin close to mid-length of forewing. Forewing with 11 apical and 6 preapical cells. Forewing venation was illustrated by Van Stalle (1991, fig. 569). Hind tibia with three lateral spines; largest located almost medially on tibia, smallest located close to femoro-tibial joint. First metatarsomere distinctly longer than second; second and third metatarsomeres almost same in size. Metatarsomeres without platellae. Metatibiotarsal formula: 5/7/7. Claws small, slightly extending arolium. Legs covered with fine setae.

***Colouration***. Body and head generally dark brown to black; carinae yellow. Pronotum yellowish to black. Legs yellowish to light brown; spines on legs apically blackish. Forewings hyaline, veins light brown to yellow, granules darker, pterostigma brown with whitish parts; apices of apical veins and veinlets slightly fuscous.

***Male genitalia***. Pygofer asymmetric, lateral sides with unique incisions and kinks; medioventral process tongue-shaped, broad. Anal segment distinctly broadened towards apex, asymmetric; apical margin with a narrow folding. Genital styli small, asymmetric; shaft moderate in size; capitulum with unique inner projections. Aedeagus largely asymmetric, basally wide, then slightly narrower, and widened towards flagellum; with two characteristic ventrobasal processes and one ventromedial process. Flagellum wide, apically membranous; processes running parallel to the flagellum, different in size and bearing unique spikes.

#### Distribution.

Luzon Island, Philippines.

#### Etymology.

The genus name is derived from a truncation of “Philippines” joined to a truncation of *Oliarus*, a reference to the type localities of known species. The name of the new genus is masculine in gender.

#### Diagnosis.

*Philippinarus* gen. nov. can be distinguished from other genera of Pentastirini by the wing venation, proportions of the body, shape of hind leg and arrangements of spines of the metatarsi (7/7, no platellae) and especially by the configuration of the male genitalia (arrangement of the ventrobasal processes of aedeagus; presence of spiny processes on the flagellum; wide anal segment and unique pygofer processes). Based on the generic concept of *Oliarus* Stål, 1862 provided by Emeljanov (2001) and later amended by Löcker et al. (2006), these genera can be distinguished from each other based on the number of lateral spines on the hind tibia with three in *Philippinarus* gen. nov. (only two lateral spines in *Oliarus*), the chaetotaxy of metatarsomeres which is 7/7 in *Philippinarus* gen. nov. (7–8 (rarely 9)/5 (rarely 6) in *Oliarus*) and the general external appearance and wing venation.

### Philippinarus granulatus

Taxon classificationAnimaliaHemipteraCixiidae

(Muir, 1924) gen. et comb. nov.

E2D219CB-7075-55AC-8954-AE5C2173D0FB

Fig. 1

Oliarus granulatus Muir, 1924: 514; Van Stalle 1991: 89.

#### Type material.

***Holotype***: • 1♂, “Mt. Limay / Luzon / Baker // 1184 // Oliarus / granulatus / 1078, Muir // BPBM ENT / 000 000 4675”. ***Allotype***: • 1♀, “Los Banos / Philippine Ids. // Coll. F. Muir / IX.15 // 1184 // Oliarus / granulatus / 1078, Muir // Allotype // BPBM ENT 000 000 4675A”. The holotype and allotype are deposited in the Entomology Collection of BPBM. The examination of the type specimens was based solely on what has been observed in photographs and illustrations.

**Figure 1.**
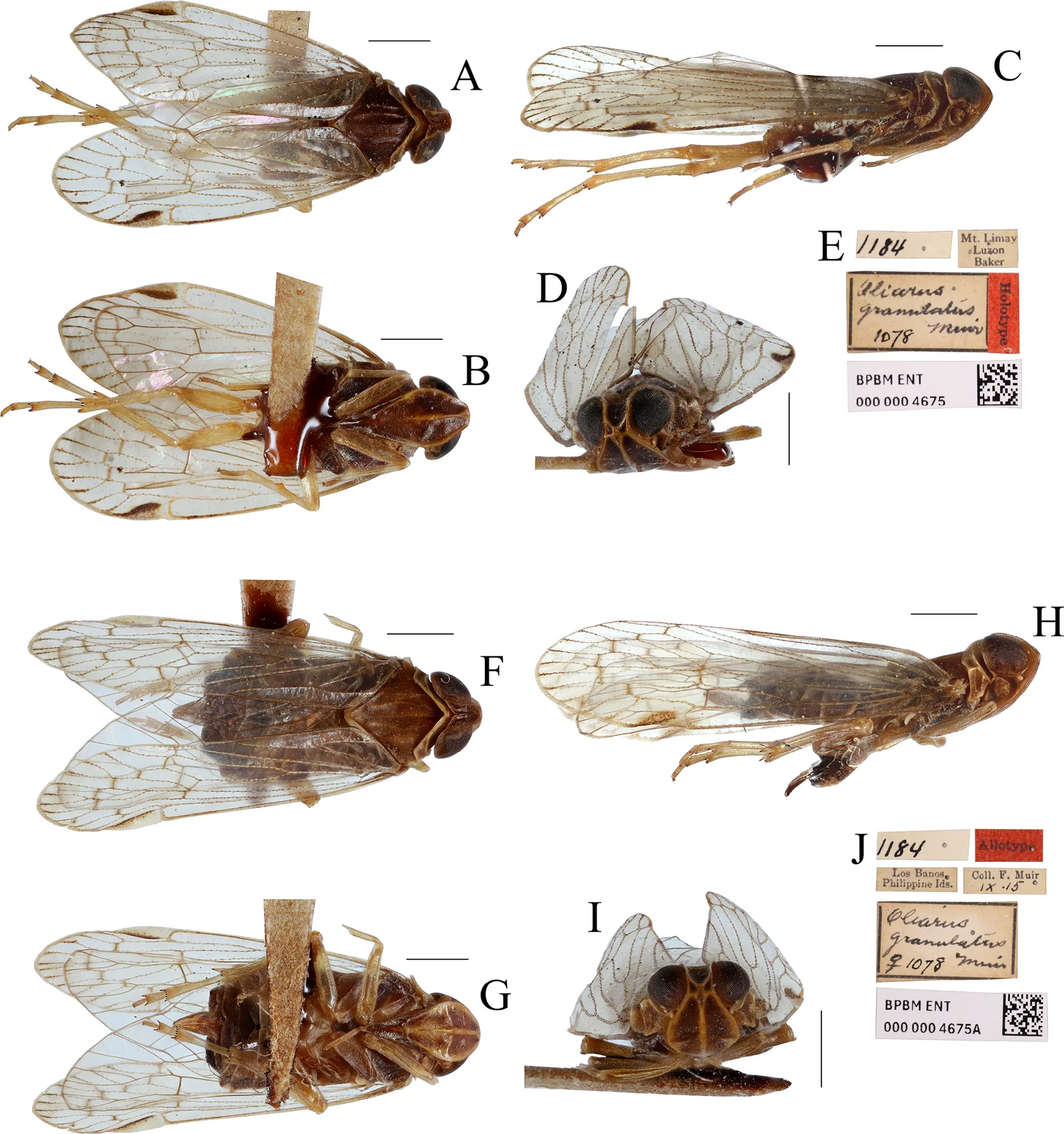
Habitus of *Philippinarus granulatus* (Muir, 1924) gen. et comb. nov., **A–E**. Holotype; **F–J**. Allotype; **A, F**. Dorsal view; **B, G**. Ventral view; **C, H**. Lateral view; **D, I**. Anterior view; **E**. Labels of holotype; **J**. Labels of allotype. Photos by Lisha Jasper, copyright belongs to the Bernice Pauahi Bishop Museum, Honolulu, Hawaii, USA. Scale bars: 1.0 mm, **E** and **J** are not to scale.

#### Diagnosis.

This species is closely related to *Philippinarus tarandiformis* gen. et sp. nov., but the two species can be easily distinguished from each other based on the following characters of the male genitalia: (1) right ventrobasal process of the aedeagus is trifurcate in *Philippinarus tarandiformis* gen. et sp. nov. (process “a” in Fig. 3A, B), while the same process in *Philippinarus granulatus* gen. et comb. nov. is bifurcate; (2) medioventral process of aedeagus with simple apex in *Ph. tarandiformis* gen. et sp. nov. (Fig. 3D), while in *Ph. granulatus* gen. et comb. nov. the same process is apically bifurcate; and (3) both apical processes of the aedeagus are covered with small spines in *Ph. tarandiformis* gen. et sp. nov. (processes “d” and “e” in Fig. 3A), while in *Ph. granulatus* gen. et comb. nov. only the longer apical process is covered with small spines.

***Total length***. Male: 5.0 mm (*N* = 1), female: 5.6 mm (*N* = 1) (after Muir 1924).

#### Distribution.

Luzon, Philippines.

#### Remarks.

The species was described by Muir (1924) based on specimens from Luzon, Philippines. Later, Van Stalle (1991) re-examined the type material and provided a lectotype designation and an amended description for the species. Figures of male genitalia were provided by Muir (1924, Pl. 1, fig. 5a, b) and Van Stalle (1991, figs 562–569), subsequently. The habitus and labels of holotype and allotype are given in Fig. 1, kindly provided by Lisha Jasper (BPBM). Based on the photos and illustrations of the species, we propose that the species should be transferred from *Oliarus* to *Philippinarus* gen. nov. based on the above-mentioned generic characters.

### Philippinarus tarandiformis gen. et sp. nov.

Taxon classificationAnimaliaHemipteraCixiidae

7DDE0C3C-7E83-5E1E-B65E-4DA5B2A197DA

https://zoobank.org/65A18973-E7D6-49A8-BF1D-967A681D5F5F

Figs 2, 3

#### Type material.

***Holotype***: • 1♂, “Philippines, N Luzon / Kalinga-AP/Abra pr. boundary / Cordillera Centr., around Pass at / 17°30'N, 121°00'E, 1600 ± 100 m / L. Dembický leg., 26–28.III.2000”, specimen card-mounted. The holotype is deposited in the Entomology Department of the MMBC.

**Figure 2.**
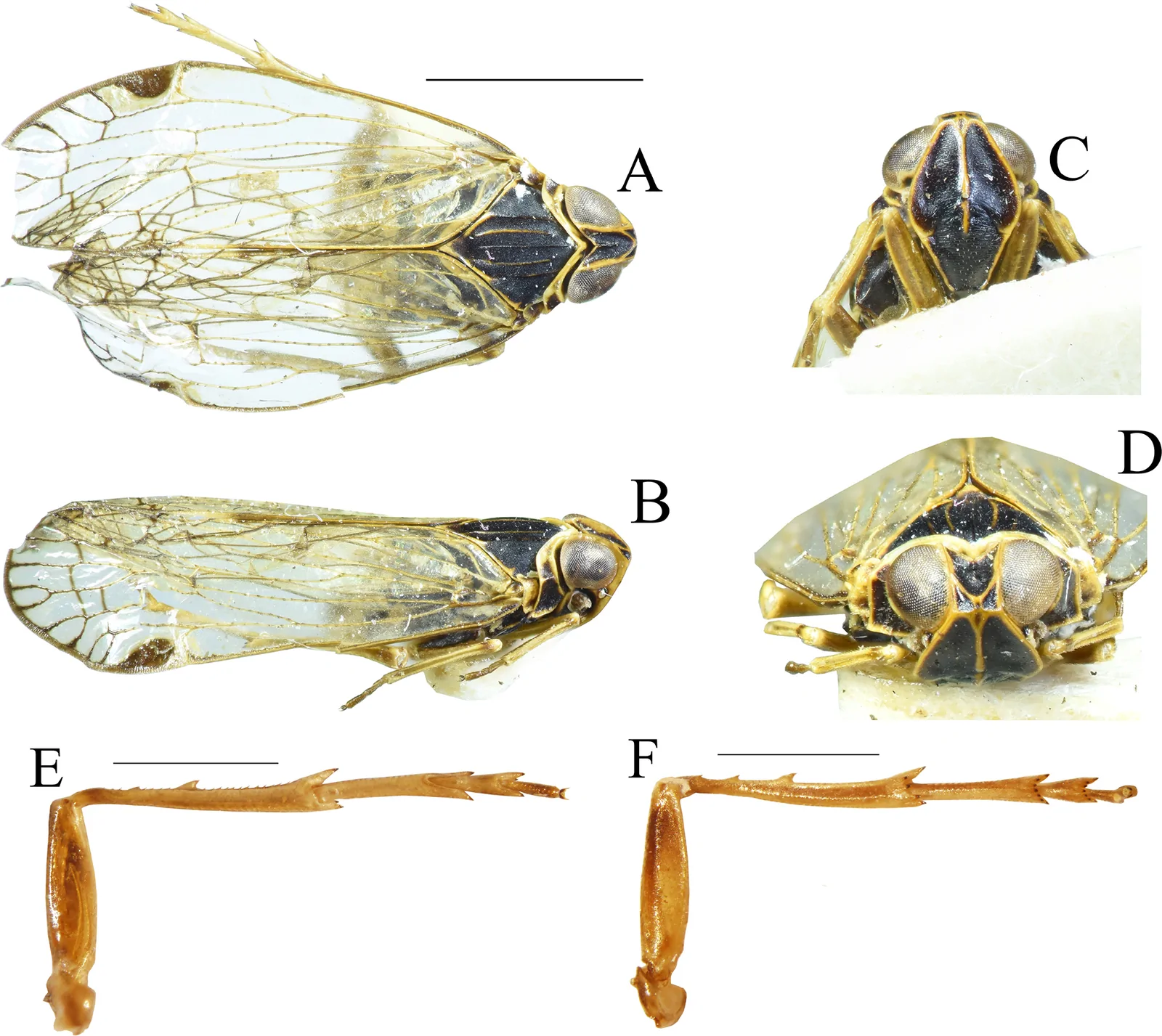
Habitus of *Philippinarus tarandiformis* gen. et sp. nov., holotype, **A–D**. Body; **E, F**. Left hind leg. **A** dorsal view; **B**. Lateral view; **C**. Frontal view; **D**. Anterior view; **E**. Dorsal view; **F**. Ventral view. Scale bars: 2 mm (**A, B**); 1 mm (**E, F**), **C** and **D** are not to scale (photos by Bence Péter Schlitt).

#### Description.

Vertex narrow, arrow-shaped, ca. 1.2× longer than wide. Frons ca. 0.4× as wide above eyes as medially; strongly widened towards epistomal suture. Pronotum thin, margins distinctly elevated. Mesonotum and scutellum depressed in lateral view. First metatarsomere ca. 1.6× as long as second (in ventral view); second metatarsomere ca. 1.3× as long as third. Metatibiotarsal formula: 5/7/7. Metatarsomeres without platellae.

***Colouration***. Vertex and foveae black; carinae yellow. Frons and clypeus black, laterally slightly dark brownish; lateral carinae yellow. Median carina of frons yellow; ocelli semi-transparent, whitish. Pronotum mostly yellowish, behind eyes with black areas; paranotal lobes black, margins yellow. Mesonotum mostly black, carinae and lateral margins with yellowish spots; scutellum yellow. Forewings transparent, costal margin brownish yellow; veins mostly yellowish, apically more brownish; granules slightly darker. Apical veinlets and apices of longitudinal veins fuscous. Pterostigma brown; around pterostigma whitish yellow. Legs entirely yellowish brown; claws and third tarsomeres darker. Spines on legs apically blackish.

**Figure 3.**
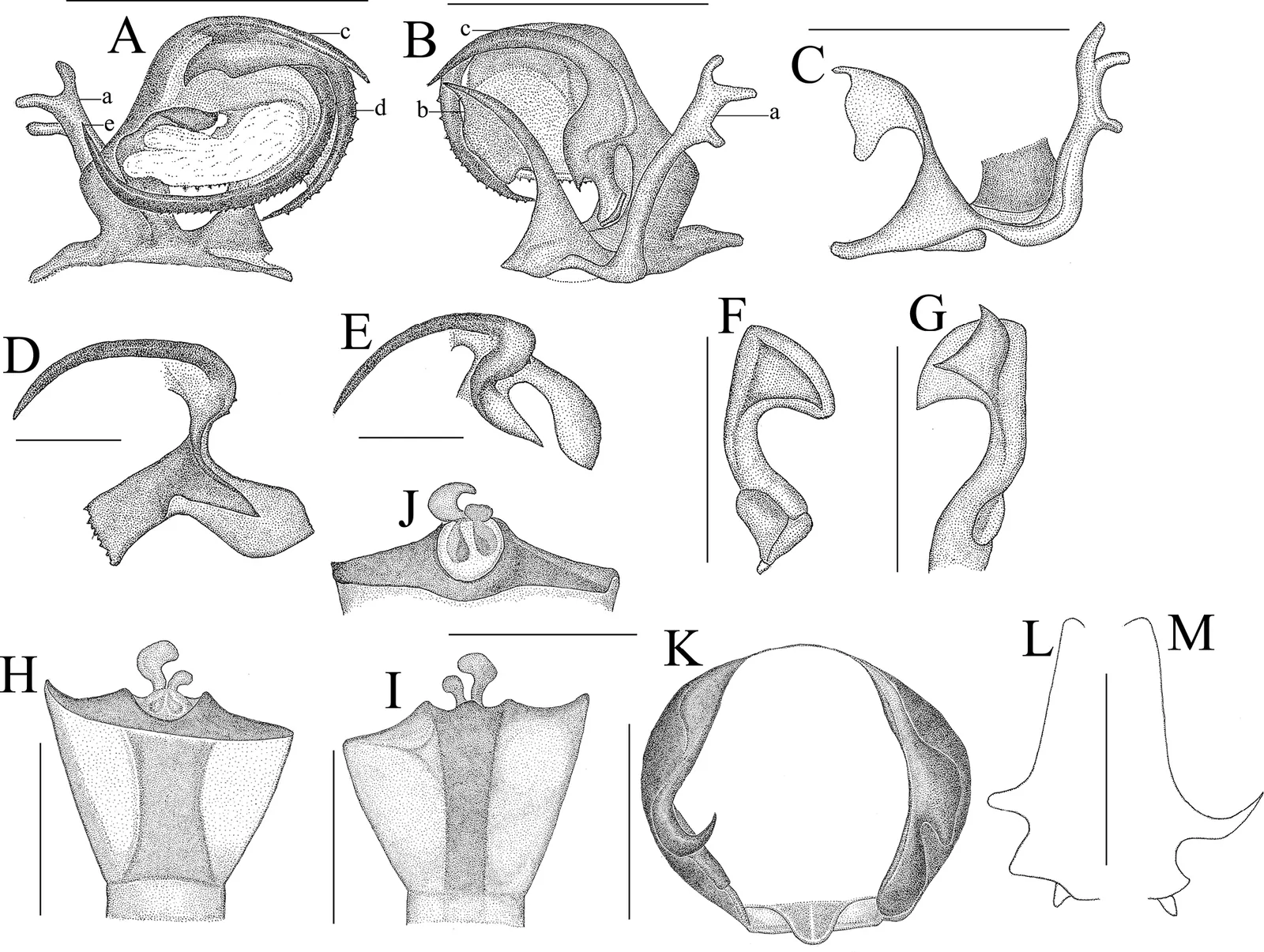
Male genitalia of *Philippinarus tarandiformis* gen. et sp. nov., holotype. **A–E**. Aedeagus; **F, G**. Genital style; **H–J**. Anal segment; **K–M**. Pygofer. **A**. Dorsal view; **B**. Ventral view; **C**. Ventrobasal processes of aedeagus in latero-ventral view; **D**. Medioventral process of the aedeagus in ventral view; **E**. Same, in ventrolateral view; **F**. Left style; **G**. Right style; **H**. Ventral view; **I**. Dorsal view; **J**. Caudal view, close-up; **K**. Caudal view; **L**. Right lateral view; **M**. Left lateral view. Scale bars: 200 μm (**D, E**); 300 μm (**F, G**); 500 μm (**A–C, H–M**) (drawings by András Orosz).

***Male genitalia***. Pygofer asymmetric; right side with a moderately deep incision, left side with a small, tapered process and incision under it in caudal view. Anal segment asymmetric, basally narrow, apically distinctly broadened; right side with longer, apically bluntly pointed part, left side shorter, more rounded. Anal segment with a narrow ventroapical folding; paraproct small, apically rounded. Genital styli asymmetric, relatively small; shaft short, curved; capitulum with unique inner lobes. Aedeagus largely asymmetric. Basal part of aedeagus widened, with two unique processes: process “a” originating right ventrobasally, slightly directed left laterad, apically widened, trifurcate, not exceeding apex of aedeagus; process “b” originating left ventrobasally, curved left laterad, apical third strongly widened, lobe-like, margins slightly undulate. Ventromedial part of aedeagus with a long, apically tapered process (process “c”) curved left laterad, slightly protruding apex of aedeagus; basally widened with characteristic lobes as in Fig. 3D, E. Aedeagus with two dorsoapical tapered processes running parallel to flagellum (process “d” and “e”), both strongly curved in ventral view, and bearing small spines; process “e” distinctly larger and longer. Flagellum wide, mostly membranous, largely asymmetrical.

***Female***. Unknown.

***Total length***. Male: 6.3 mm (*N* = 1).

#### Distribution.

Luzon, Philippines.

#### Etymology.

The specific epithet *tarandiformis*, meaning “reindeer-like”, refers to the unique antler-like right ventrobasal process (process “a”) of the aedeagus.

#### Diagnosis.

The species is closely related to *Philippinarus granulatus* (Muir, 1924) gen. et comb. nov. Differences are discussed above.

### Genus Pteroprocessus gen. nov.

Taxon classificationAnimaliaHemipteraCixiidae

1393DDA6-1945-5D78-8E08-E9883F29F182

https://zoobank.org/5ECC4F2C-4E17-4D66-9E1C-FBFF6961D1A3

#### Type species.

*Pteroprocessus nabhitabhatai* gen. et sp. nov., designated here.

#### Description.

Large planthoppers (7.2–18(?) mm). Head in dorsal view narrower than pronotum. Vertex broad, apically slightly narrower; subapical transverse carina convex, posterior margin deeply concave. Median carina of vertex reaching or almost reaching anterior margin. Eyes large, longer than wide; lateral ocelli large. Dorsolateral foveae moderate size, roughly quadrate, posteriorly elongate, tapered; dorsoapical fovea small, subquadrate. Frons narrower above eyes, enlarged towards postclypeus, widest below eyes; lateral margins slightly elevated. Frons with distinct median carina and moderately distinct sublateral carinae. Median carina on frons forked below apical transverse carina defining a distinct, triangular frontal fovea; median carina continuous throughout face, reaching apex of anteclypeus. Fenestrae and median ocellus well-developed. Epistomal suture convex. Postclypeus slightly narrowing towards anteclypeus; with visible transverse alternating striation; margins slightly elevated. Anteclypeus tapered. Rostrum projects beyond hind coxae. Pedicel large, globular; antennal flagellum thin, moderate in length. Pronotum thin, posterior margin deeply concave; margins elevated; widened behind eyes. Median carina of pronotum distinct. Paranotal lobes large, leaf-shaped, apically tapered, with transverse carina close to apex; margins distinctly elevated. Mesonotum large, almost as long as wide, with five carinae; lateral carinae slightly convex, intermediate carinae incomplete; median carina straight, not reaching tip of scutellum. Scutellum indistinct. Mesonotum in lateral view flattened. Tegulae large, kidney-shaped. Macropterous. Forewings extend the abdomen by an entire length of it. Pterostigma large, elongate, distinct. Forewings with small granules along veins, each bearing small setae. Forewings with moderate size ScP + R, forking into ScP + RA and RP basad of CuA fork; RA with three terminals; RP stem long, ca. 1.6× as long as ScP + RA stem, with three terminals, RP_2_ forking into two branches; *ir* veinlet present between forking of RA and last terminal of RP, shorter than *rp-mp* and *rp-mp’*; *rp-mp* present between forking of MP and slightly distad of midpoint of RP stem; *rp-mp’* present apicad between forking of RP and last terminal of MP_1_. M stem very long, forking well distad of ScP + RA and CuA forks, nearly at level of apex of clavus. M with two main branches forking into five terminals, M_1_ forking into two branches; two *im* veinlets present, *im’* slightly shorter. CuA stem longer than stem of ScP + R, forking distad of ScP + R, slightly basad of midpoint of MP. CuA_1_ and CuA_2_ unforked; *icua* and *icu* present, *icua* shorter. Pcu and A_1_ fused distad of midpoint of clavus; Pcu + A_1_ shorter than A_1_, reaching anal margin at almost mid-length of forewing. Forewings with 11 apical and 6 preapical cells. Hindwings with small, barely visible *wca*; *r-m* long; *m-cua* oblique; connection between MP and CuA V-shaped. Hind tibiae with 5–8 conspicuously small lateral spines. First metatarsomeres longer than second, second metatarsomeres longer than third. Metatarsomeres without platellae. Metatibiotarsal formula: 6/7/5. Claws small, slightly extending arolium. Legs covered with fine setae.

***Colouration***. Generally brown to yellowish or dark brown to blackish; carinae paler. Pronotum lighter than head and mesonotum. Legs yellowish to brown; spines on legs apically blackish. Forewings semi-transparent with unique markings and colouration; veins and granules brown to black. Hind wings apically fuscous.

***Male genitalia***. Pygofer narrow, asymmetric laterally, without conspicuous processes. Medioventral process large, longer than wide, tongue-shaped; with a distinct ventral carina and with unique, symmetric or asymmetric ventrolateral appendages. Anal tube slightly or largely asymmetric, distinctly longer than wide, dorsally moderately or distinctly tubularly elevated; paraproct long. Anal tube ventrally with unique lobe. Genital style moderately asymmetric, cane-shaped. Aedeagus with a distinct ventrobasal process “a”; shaft thin, without or with processes. Flagellum coiled, with characteristic processes.

***Female***. Larger than male. Anal segment large and wide, rounded; wax plate laterally thickened. Pregenital sternite excavated medially. Ovipositor orthopteroid-type according to classification by Bourgoin (1993). Gonoplac elongate, triangular, basally broadened. Gonapophysis VIII basally broadened, finger-like, apically filiform; gonapophysis IX filiform.

#### Distribution.

Thailand: Trang Province; China: Yunnan Province; Myanmar: Rangoon.

#### Etymology.

The generic name is derived from the Greek *pteron* (“wing”) and the Latin *processus* (“process”), referring to the unique wing-like processes on the medioventral process of the pygofer. The genus name is masculine in gender.

#### Diagnosis.

*Pteroprocessus* gen. nov. can be distinguished from other genera of Pentastirini by wing venation, proportions of the body, shape of hind legs and configuration of male genitalia (cane-shaped genital styli; presence of a characteristic ventrobasal process; aedeagal shaft relatively thin, flagellum coiled; pygofer narrow and asymmetric). Arrangements of spines of the metatarsi (7/5, no platellae) seem to be common among Oriental Pentastirini, especially *Oliarus* s.str., however, the conspicuously small lateral spines on the hind tibiae are unique and clearly separate it from the genus *Oliarus*.

### Pteroprocessus binghami

Taxon classificationAnimaliaHemipteraCixiidae

(Distant, 1911) gen. et comb. nov.

6ABE2614-FFCB-5DD4-965B-CB6236CAA7B1

Oliarus binghami Distant, 1911: 738.
Indolipa binghami (Distant, 1911) in Emeljanov (2001): 72.

#### Diagnosis.

The species was described from Rangoon based on a single female specimen, and no male material is currently known from the type locality. Therefore, comparisons are limited to characters of the female terminalia and external characters, which makes the comparison difficult. Based on the markings of the forewing and general morphology of the female terminalia, the species is closely related to *Pteroprocessus nabhitabhatai* gen. et sp. nov. Illustrations of Van Stalle (1991) suggest differences in the basal portion of gonapophysis VIII, as well as the total length of the body. However, the significance of these differences remains uncertain. The identity of *P. binghami* (Distant, 1911) gen. et comb. nov. can only be resolved through examination of male specimens from the type locality and subsequent comparison of their genital morphology with that of *P. nabhitabhatai* gen. et sp. nov.

***Total length***. 18 mm (after Distant 1911, most probably erroneous measurement).

#### Distribution.

Myanmar, Rangoon.

#### Remarks.

The species was described by Distant (1911) as *Oliarus binghami* from Rangoon, Myanmar. Later, Van Stalle (1991) re-examined the type material and provided a lectotype designation and an amended description for the species (Van Stalle 1991, figs 625–628). Recently, Emeljanov (2001) transferred the species to the genus *Indolipa* Emeljanov, 2001. Here, based on the figures and description provided by Van Stalle (1991), especially the wing venation and markings as well as the general colouration and morphology of female terminalia, we propose that the species should be transferred from *Indolipa* to *Pteroprocessus* gen. nov.

### Pteroprocessus nabhitabhatai gen. et sp. nov.

Taxon classificationAnimaliaHemipteraCixiidae

4DB5A48C-54CC-5D38-A928-8C42A4546269

https://zoobank.org/42EB578E-9AA2-4FDE-8841-58D8BF75F962

Figs 4, 5, 6, 7, 8

#### Type material.

***Holotype***: • 1♂, “Thailand, Trang Prov. / Khao Chong Bot. G. [= Botanical Garden] by light / at the center, 21.XI.2003 / leg. A. Orosz & Gy. Sziráki”. ***Paratypes***: • 3♂♂, same label data as the holotype; • 2♂♂ “Thailand, Prov. Trang / Thung Khai Bot. G. [= Botanical Garden], rainforest / 19.XI.2004 leg. M. Földvári / A. Orosz, L. Papp”; • 1♀ “Thailand, Trang Prov. / Khao Chong Bot. G. at the / staff center, 20.XI.2003 / leg. A. Orosz & Gy. Sziráki”. The holotype and all paratypes are card-mounted. The holotype and 4 paratypes are deposited in the Hemiptera Collection of the HNHM, while 2 male paratypes from Khao Chong are deposited in the Entomology Collection of THNHM.

**Figure 4.**
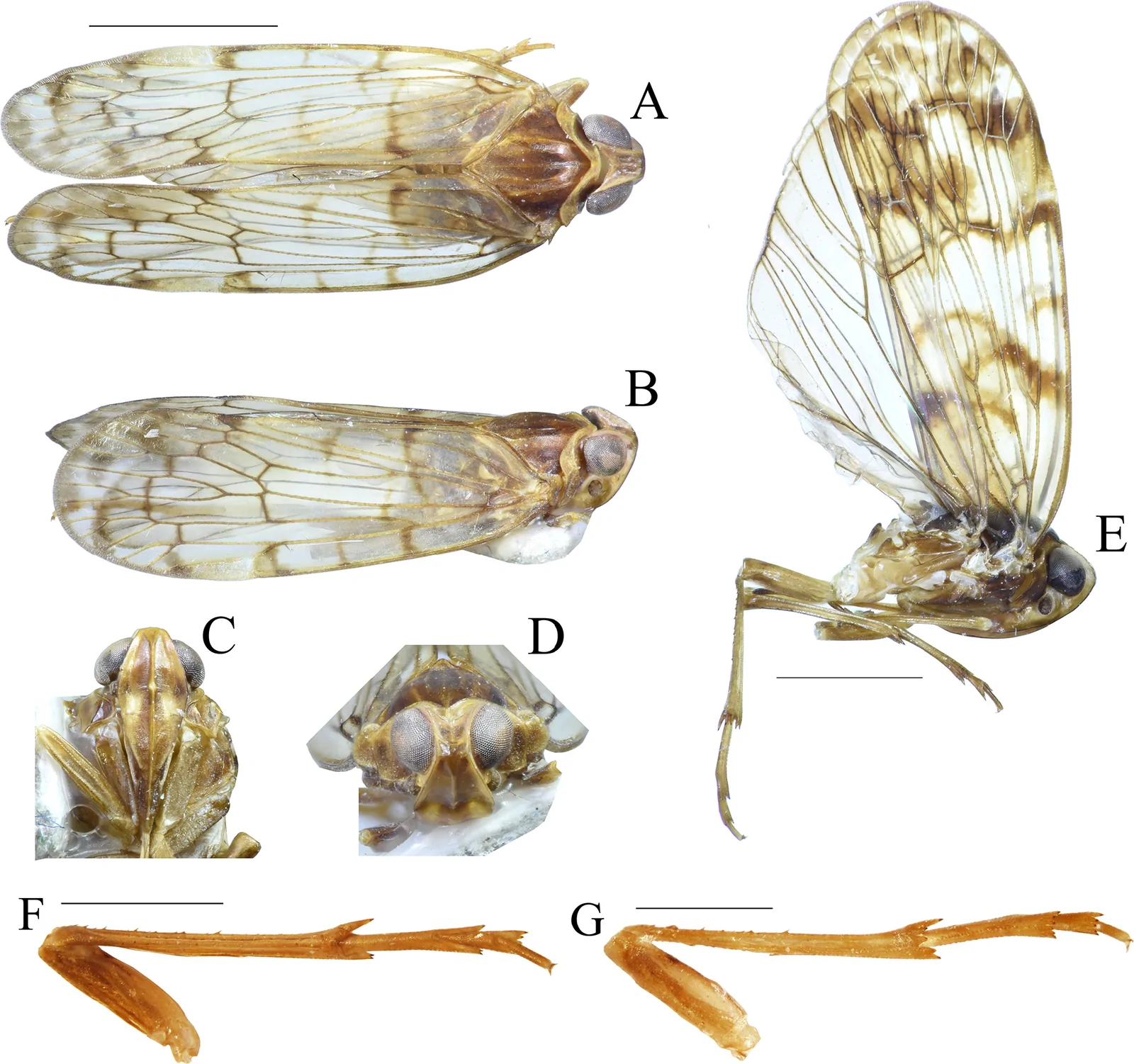
Habitus of *Pteroprocessus nabhitabhatai* gen. et sp. nov., **A–D**. Holotype; **E**. Female paratype in lateral view; **F, G**. Left hind leg. **A**. Dorsal view; **B**. Lateral view; **C**. Frontal view; **D**. Anterior view; **F**. Dorsal view; **G**. Ventral view. Scale bars: 2.0 mm (**A, B, E**); 1.0 mm (**F, G**), **C** and **D** are not to scale (photos by Bence Péter Schlitt).

**Figure 5.**
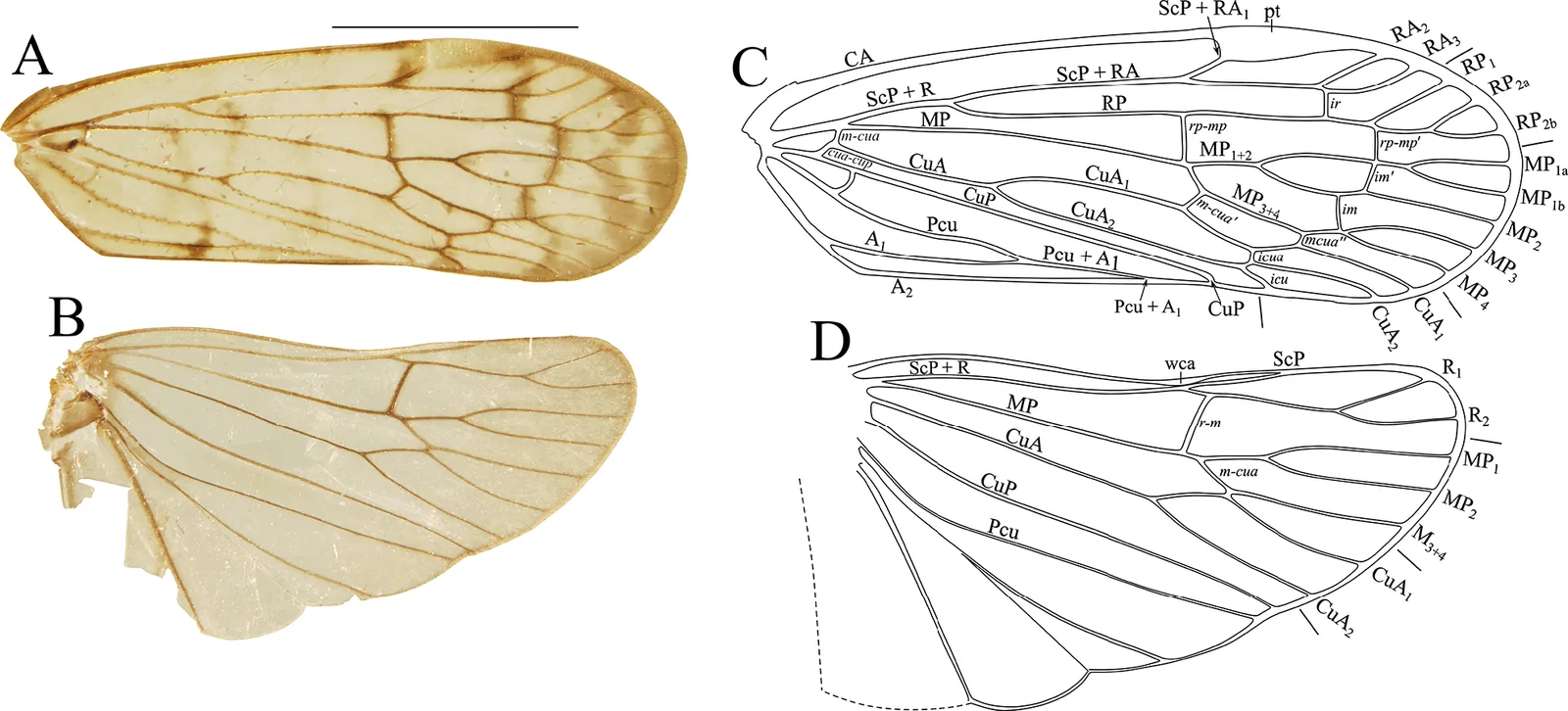
Left fore- and hindwing of *Pteroprocessus nabhitabhatai* gen. et sp. nov. **A**. Forewing; **B**. Hindwing; **C**. Forewing venation pattern; **D**. Hindwing venation pattern. Scale bar: 2.0 mm, **C, D** not to scale (photos and drawings by Bence Péter Schlitt).

#### Description.

Vertex slightly wider than long, with complete median carina. Frons ca. 0.5× as wide above eyes as medially, widened towards epistomal suture. Pronotum thin, margins elevated. Mesonotum and scutellum depressed in lateral view. First metatarsomere ca. 1.6× as long as second (in ventral view); second metatarsomere ca. 1.3× as long as third. Metatibiotarsal formula: 6/7/5. Hind tibiae with 5–7 small lateral spines. Metatarsomeres without platellae.

***Colouration***. Vertex and frons mostly light brownish with yellowish areas. Carinae of foveae more yellowish, medially brownish. Frons slightly darker brownish around fenestrae. Frons with distinct, white maculae close to epistomal suture and expanded to postclypeus. Median ocellus semi-transparent, whitish. Clypeus brown with yellowish spots; striation darker brownish; median carina yellow. Rostrum dark brown apically. Pronotum, paranotal lobes and tegulae yellowish. Mesonotum and scutellum brown with lighter or darker spots. Forewings mostly semi-transparent; veins mostly brownish, veinlets darker. Forewings with unique brownish fasciae and markings. Hind wings transparent with dark veins; apical cells slightly fuscous. Hind legs yellowish; forelegs, claws and third metatarsomere slightly darker. Spines on legs apically black.

**Figure 6.**
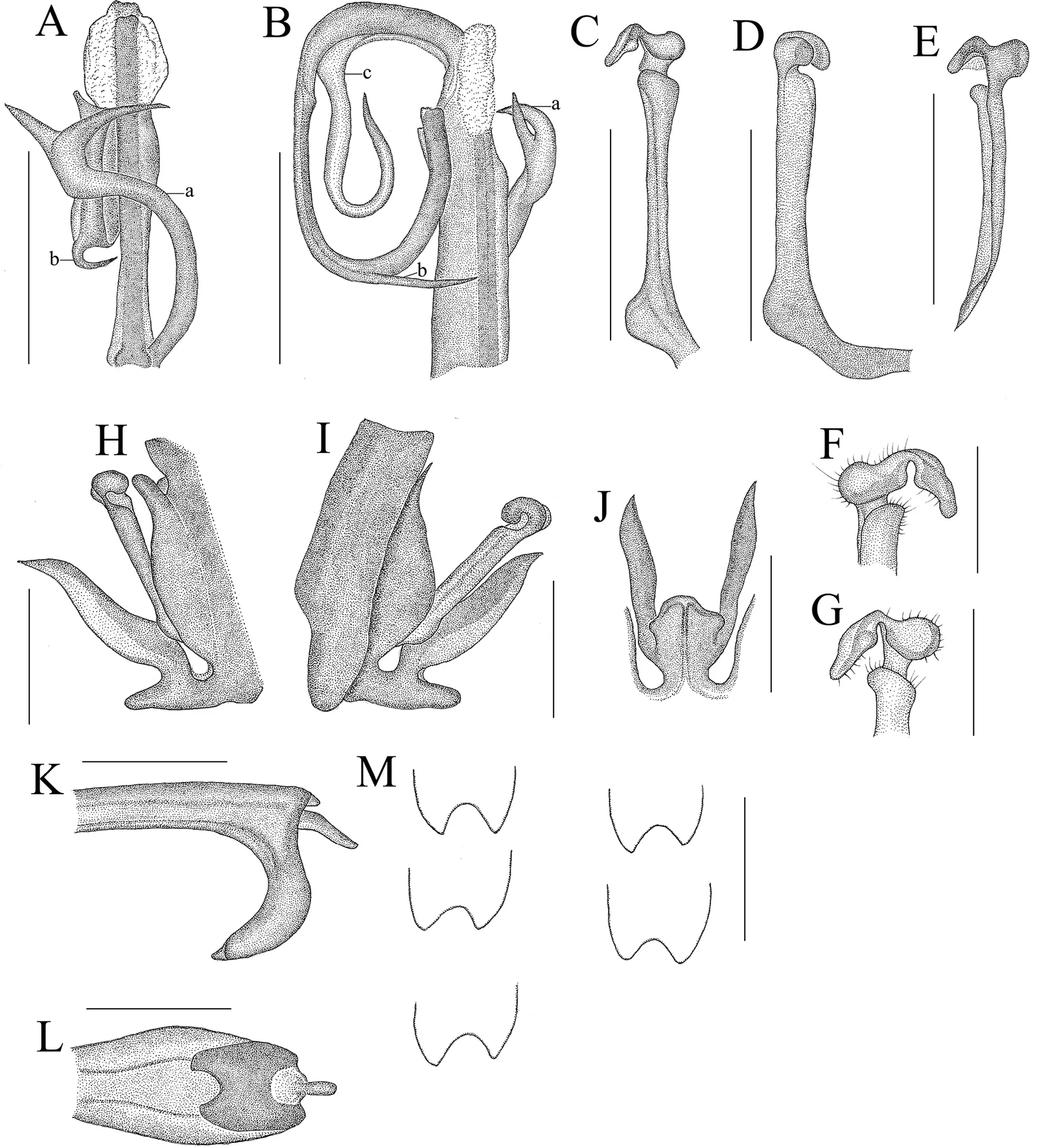
Male genitalia of *Pteroprocessus nabhitabhatai* gen. et sp. nov. **A, B**. Aedeagus; **C–G**. Genital styli; **H, I**. Apex of pygofer without aedeagus and anal segment; **K–M**. Anal segment; **A**. Ventral view; **B**. Left lateral view; **C**. Left style, lateral view; **D**. Right style, lateral view; **E**. Right style, caudal view; **F**. Capitulum of right style; **G**. Capitulum of left style; **H**. Left lateral view; **I**. Right lateral view; **J**. Medioventral process of pygofer; **K**. Right lateral view; **L**. Ventral view; **M**. Varieties of margin of anterior lobe of anal segment. Scale bars: 200 μm (**F, G**); 500 μm (**A–E, H–M**) (drawings by András Orosz).

***Male genitalia***. Pygofer asymmetric, relatively narrow; left side with apically acute portion, while right side with finger-like apical portion and broader medially. Medioventral process of pygofer with two, large and moderately wide, gladiate, ventrolateral appendages spanning 2/3 of pygofer; appendages of medioventral process apically slightly tapered. Anal tube elongate, distinctly longer than wide, dorsally tubularly elevated, laterally slightly thickened; paraproct moderately long, protruding beyond anal tube. Anal tube ventrally with medially incised, median lobe of moderate length. Median lobe on anal tube often asymmetric, margin slightly varying as in Fig. 6M. Genital styli moderately asymmetric, almost reaching upper part of pygofer, cane-shaped; shaft remarkably long and thin, basally with widened kink in lateral view; capitulum asymmetric, wider than long, with globular and lobe-like portions as in Fig. 6F, G. Aedeagus asymmetric; shaft thin and long. Aedeagus ventrobasally with a distinct, elongate and undulate, apically bifurcated process (process “a”). Flagellum coiled, moderately thin, with two processes: process “b” originating before apex of flagellum, directed ventrad, thin, apically tapered and process “c” originating in apical third of flagellum, lyre-shaped, tapered apically.

**Figure 7.**
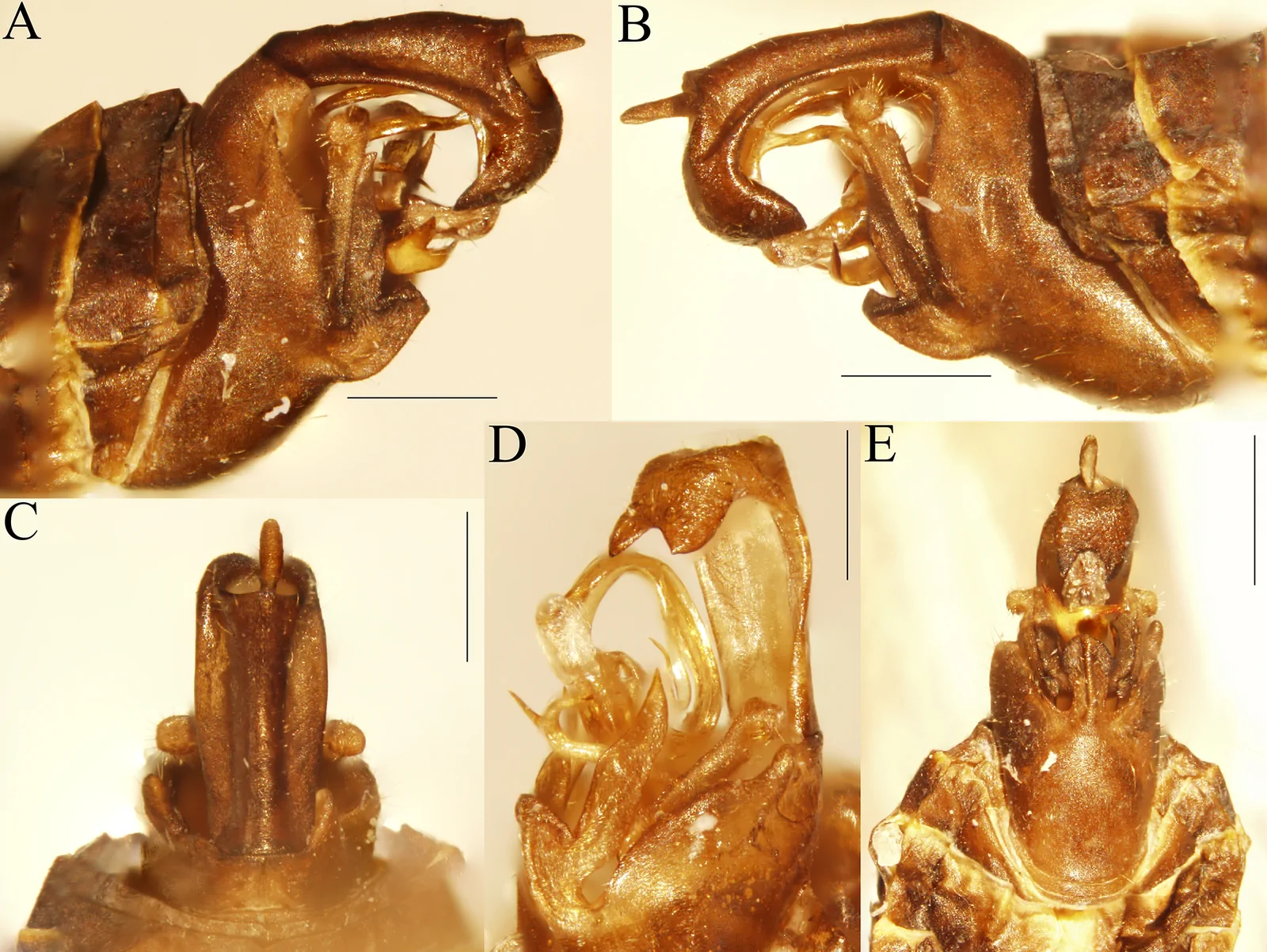
Male terminalia of *Pteroprocessus nabhitabhatai* gen. et sp. nov., paratype. **A**. Left lateral view; **B**. Right lateral view; **C**. Dorsal view; **D**. Latero-caudal view; **E**. Ventral view. Scale bars: 1 mm (photos by Bence Péter Schlitt).

**Figure 8.**
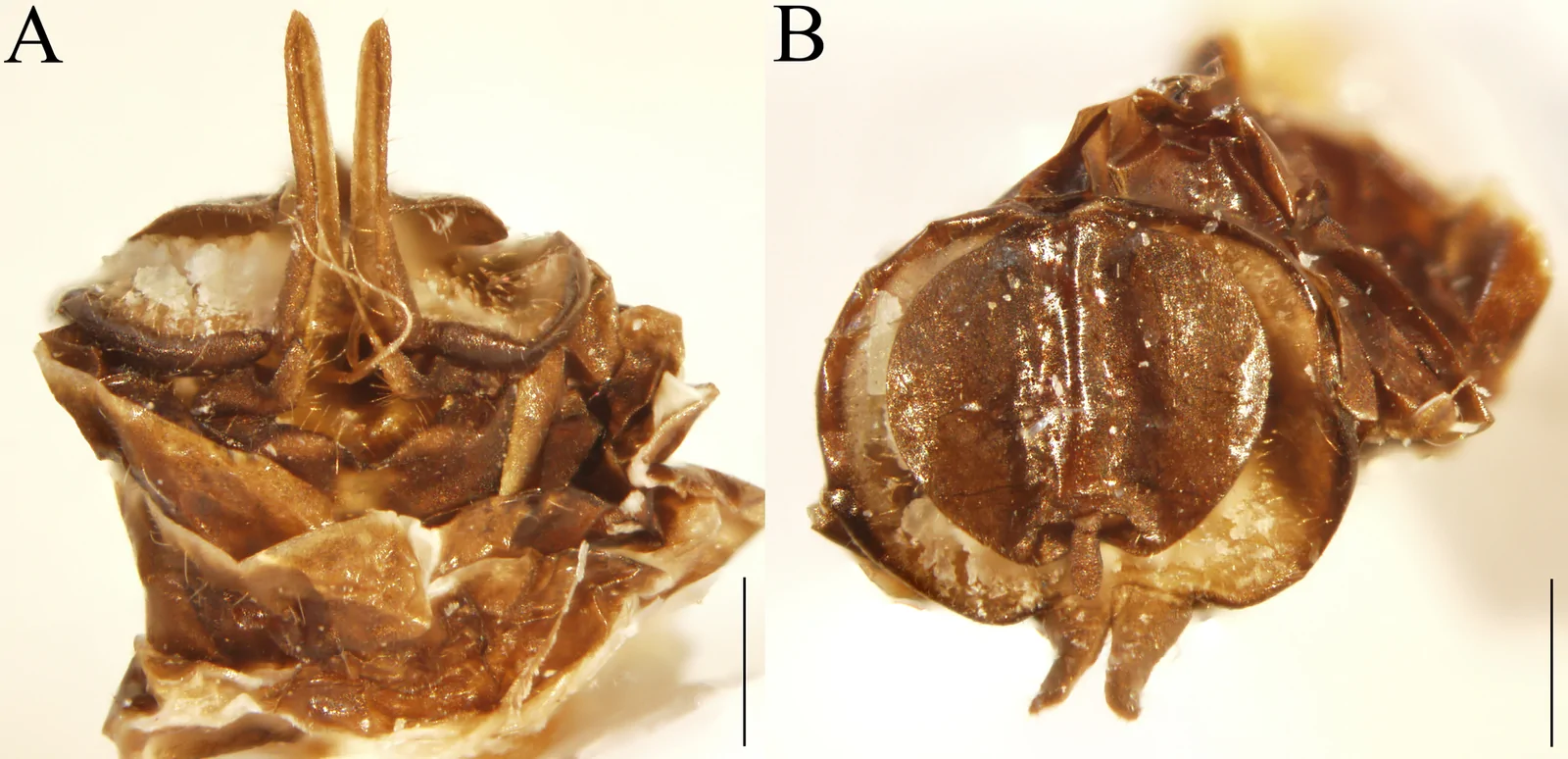
Female terminalia of *Pteroprocessus nabhitabhatai* gen. et sp. nov., paratype. **A**. Ventral view; **B**. Caudal view. Scale bars: 1 mm (photos by Bence Péter Schlitt).

***Female***. Larger than male. Female terminalia as in the generic description.

***Total length***. Male: 7.2–7.5 mm (*N* = 6); Female: 8.5 mm (*N* = 1).

#### Distribution.

Thailand, Trang Province.

#### Etymology.

The species is dedicated to Dr Jarujin Nabhitabhata (1949–2008), a prominent Thai biologist and zoologist, former director of the THNHM. The specific epithet is a proper noun in the genitive case.

#### Diagnosis.

The species is closely related to *Pteroprocessus binghami* (Distant, 1911) gen. et comb. nov. based on the general morphology of the female terminalia and markings of the forewings. The problematic comparison of the two species is discussed above.

### Pteroprocessus obscurinervis gen. et sp. nov.

Taxon classificationAnimaliaHemipteraCixiidae

A7A72777-0275-53F5-BF43-9927FC3E6FDE

https://zoobank.org/EB859C82-3212-42B8-997E-EF97EBC4296D

Figs 9, 10, 11

#### Type material.

***Holotype***: • 1♂, “China, Yunnan, 15 km SE of / Mohan, env. of Miaozhai, 1236 m / 30–31.07.2017. at light / leg. D. Rédei & J. F. Tsai”, specimen card-mounted. ***Paratype***: • 1♂, same label data as the holotype, specimen card-mounted. The holotype and paratype are deposited in the Hemiptera Collection of the HNHM.

**Figure 9.**
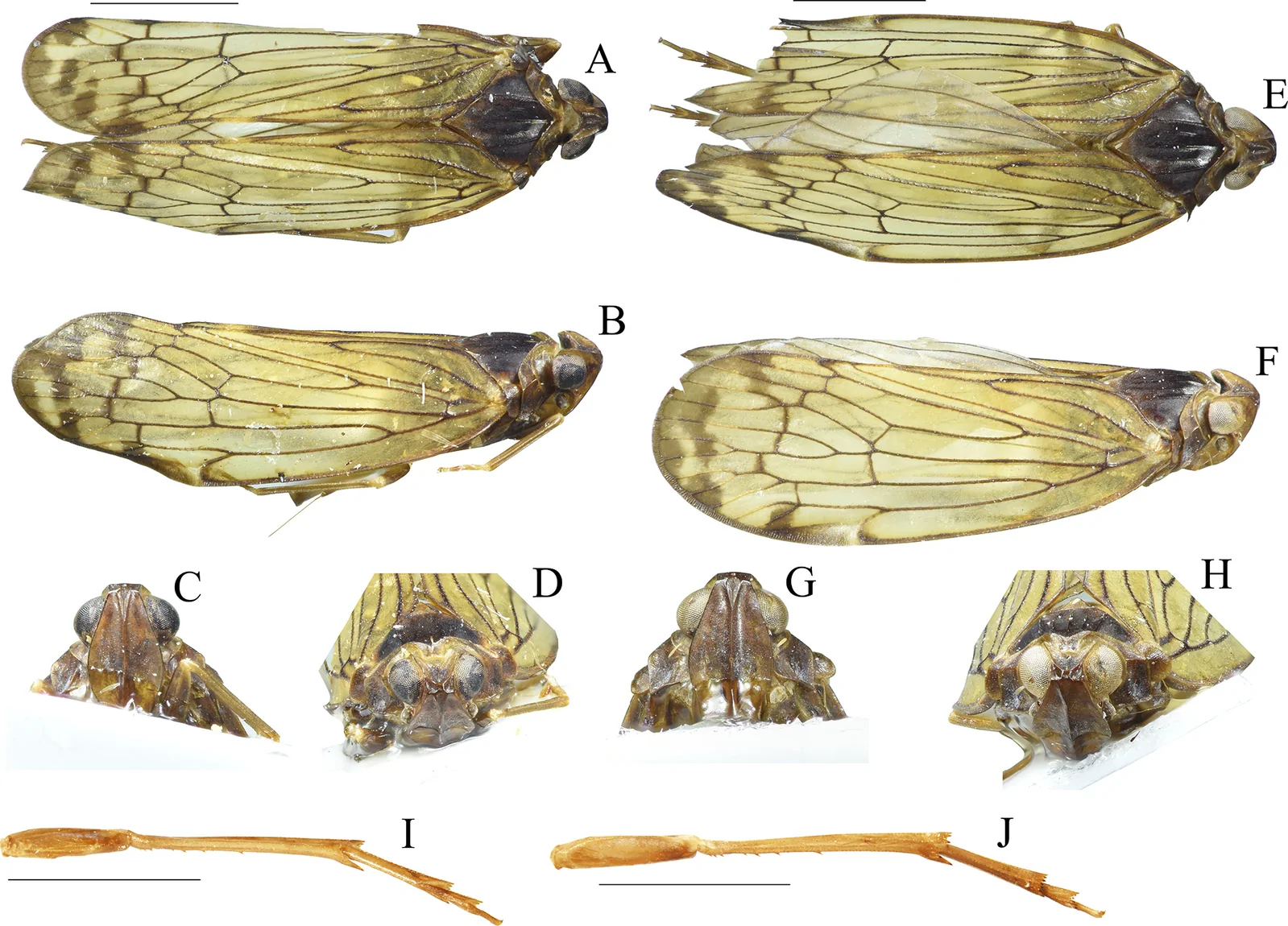
Habitus of *Pteroprocessus obscurinervis* gen. et sp. nov., **A–D**. Holotype; **E–H**. Male paratype; **I, J**. Right hind leg. **A**. Dorsal view; **B**. Lateral view; **C**. Frontal view; **D**. Anterior view; **E**. Dorsal view; **F**. Lateral view; **G**. Frontal view; **H**. Anterior view; **I**. Dorsal view; **J**. Ventral view. Scale bars: 2.0 mm, **C, D** and **G, H** are not to scale (photos by Bence Péter Schlitt).

#### Description.

Vertex wider than long, median carina almost reaching anterior margin of vertex. Frons ca. 0.4× as wide above eyes as medially, widened towards epistomal suture. Pronotum thin, margins elevated. Mesonotum and scutellum depressed in lateral view. In ventral view, first metatarsomere ca. twice as long as second; second metatarsomere ca. 1.2× as long as third. Metatibiotarsal formula: 6/7/5. Hind tibiae with 6–8 small lateral spines. Metatarsomeres without platellae.

**Figure 10.**
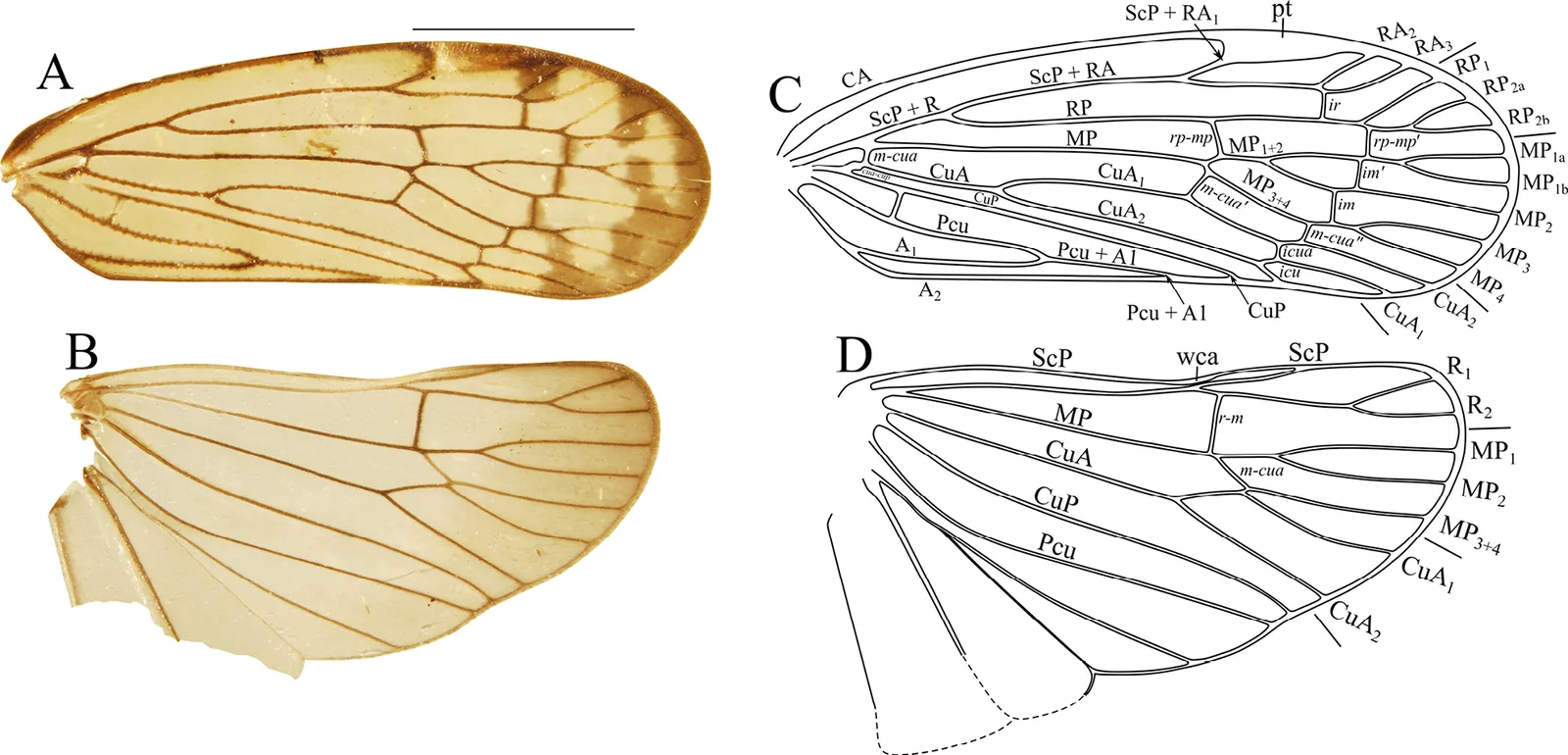
Left fore- and hindwing of *Pteroprocessus obscurinervis* gen. et sp. nov. **A**. Forewing; **B**. Hindwing; **C**. Forewing venation pattern; **D**. Hindwing venation pattern. Scale bar: 2.0 mm; **C, D** are not to scale (photos and drawings by Bence Péter Schlitt).

***Colouration***. Vertex, frons and clypeus mostly dark brown with paler areas; margins and carinae slightly darker, blackish. Frons slightly lighter around fenestrae. Median ocellus semi-transparent, whitish. Striation of clypeus distinct, darker. Rostrum brown to yellowish, apically blackish. Pronotum, paranotal lobes and tegulae dark brown with lighter spots. Mesonotum mostly black with lighter brownish spots close to lateral margins and scutellum; scutellum brownish. Forewings pale yellow, semi-transparent with blackish markings apically as in Fig. 10A; veins (including veinlets) and granules distinctly black to dark brown. Hind wings semi-transparent, apically fuscous; veins dark brown to blackish. Legs brownish with lighter spots; claws darker. Spines on legs apically black.

**Figure 11.**
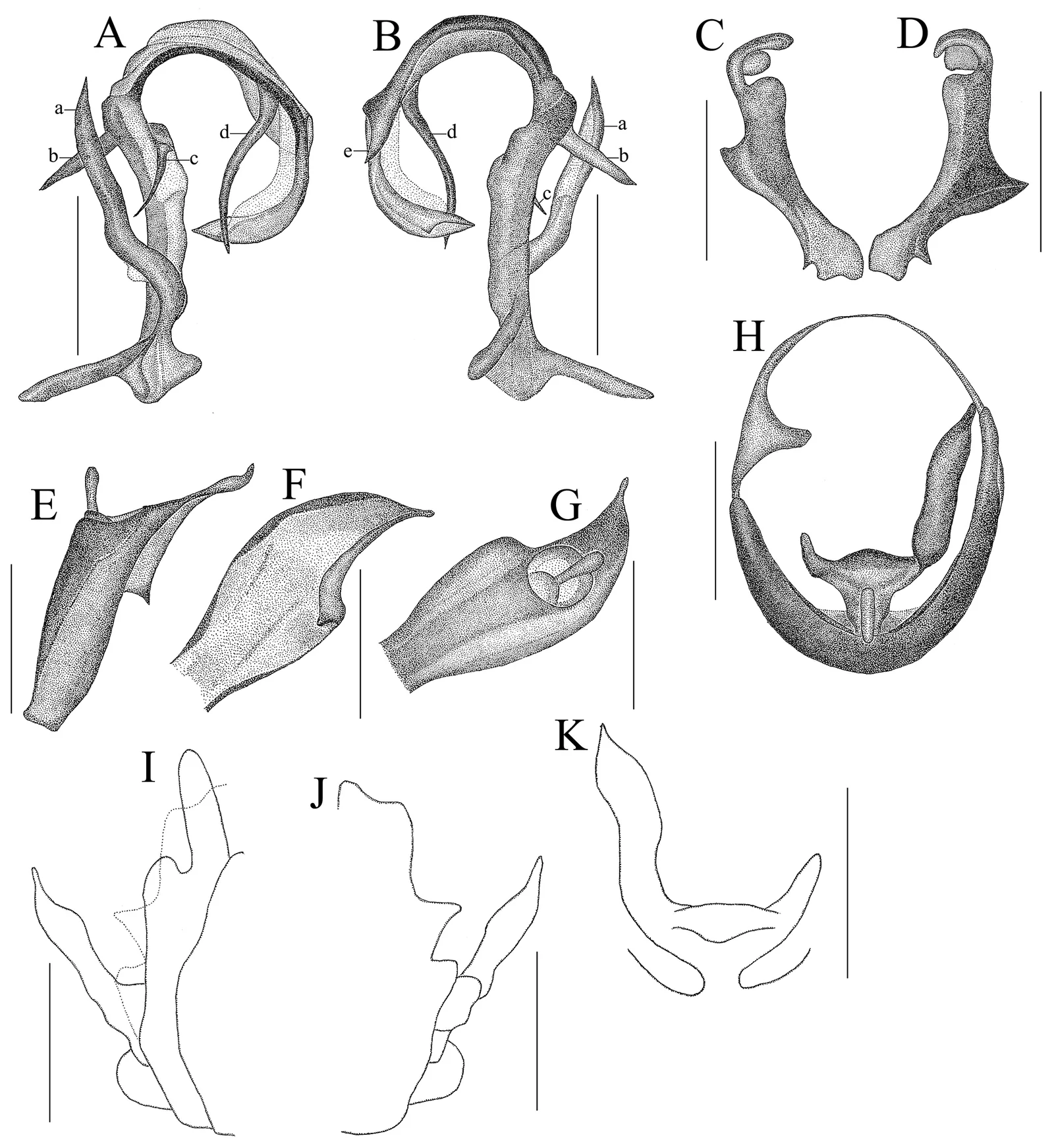
Male genitalia of *Pteroprocessus obscurinervis* gen. et sp. nov. **A, B**. Aedeagus; **C, D**. Genital styli; **E–G**. Anal tube; **I, J**. Apex of pygofer without aedeagus and anal segment. **A**. Right lateral view; **B**. Left lateral view; **C**. Right style; **D**. Left style; **E**. Lateral view; **F**. Ventral view; **G**. Dorsal view; **H**. Medioventral process in caudal view; **I**. Right lateral view; **J**. Left lateral view; **K**. Medioventral process in inner view. Scale bars: 500 μm (drawings by András Orosz).

***Male genitalia***. Pygofer asymmetric, relatively narrow; left side with apical excavation and small kink above; right side with an elongate, finger-like apical portion. Medioventral process largely asymmetric, right ventrolateral appendage long, undulate, apically tapered, left appendage rudimentary. Anal tube asymmetrical, moderately elongate; medially slightly tubulate. Anterior margin of anal tube acuate with a small ventral lobe as in Fig. 11F; paraproct cone-shaped. Genital styli asymmetric; shaft relatively short, basally with different sized kinks; capitulum wider than long, with asymmetric globular and lobe-like portions as in Fig. 11C, D. Aedeagus asymmetric; shaft thin with three processes: process “a” large, moderately wide, slightly undulating, originating right laterobasally, running parallel with shaft ventrally and directed caudad; process “b” moderate in size, wide, apically tapered and originating ventroapically, directed ventrad or caudad; process “c” short and thin, apically tapered, originating right laterally, curved ventrad. Flagellum coiled, apex not reaching shaft, with two processes. Process “d” long, base slightly widened, apex tapered; originating almost medially on flagellum, directed cephalad; process “e” originating slightly after mid-length of flagellum, basally distinctly widened, with a median kink; apically tapered and slightly curved dorsad. Flagellum slightly broadened, apically acuate.

***Female***. Unknown.

***Total length***. Male: 8.5–8.7 mm (*N* = 2).

#### Distribution.

China, Yunnan Province.

#### Etymology.

The specific epithet *obscurinervis*, meaning “dark-veined”, refers to the characteristic darkened wing venation of the species.

#### Diagnosis.

The species is unique within the genus and clearly differs from all known congeners based on the colouration and marking on the forewings and the morphology of the male terminalia.

## Discussion

**Figure 12.**
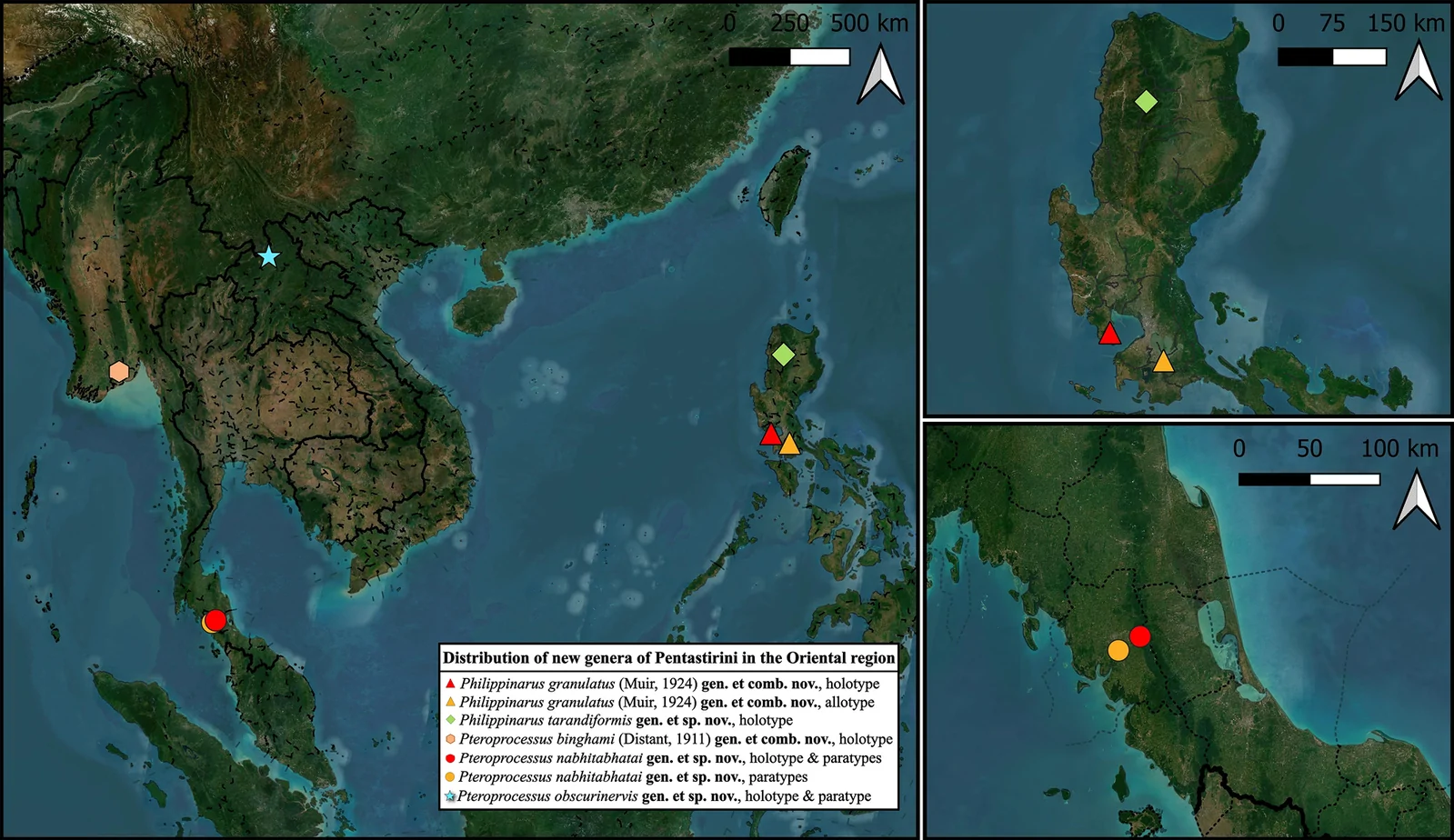
Distribution of the newly established genera and species in the Oriental Region. Made with QGIS, Basemap: MapTiler Satellite Plain (available from: https://www.maptiler.com/maps/satellite/).

The genus *Philippinarus* gen. nov. is currently restricted to Luzon Island, while *Pteroprocessus* gen. nov. seems to be more widely distributed across the Indochinese Peninsula and into China (Fig. 12). Further representatives are expected to be found, possibly even greatly extending the distribution range.

The Oriental Pentastirini exhibit a remarkable diversity. Despite previous work on this group, the fauna of the region remains largely unknown and needs further faunistic and taxonomic investigations. Despite previous taxonomic approaches to the generic placement of the group, some genera remain polyphyletic and more detailed taxonomical and phylogenetic investigations are needed to reveal the true relationships among known species and genera, as previously emphasized by Löcker et al. (2006). Because the group’s generic concepts need revision due to paraphyletic genera (e.g., *Oliarus*, *Indolipa*), we cannot provide a useful key to separate the currently known genera of Pentastirini from the region. However, the distinct and unique morphology of the newly described taxa provides reliable generic distinction between other members of Pentastirini.

## Supplementary Material

XML Treatment for Philippinarus

XML Treatment for Philippinarus granulatus

XML Treatment for Philippinarus tarandiformis

XML Treatment for Pteroprocessus

XML Treatment for Pteroprocessus binghami

XML Treatment for Pteroprocessus nabhitabhatai

XML Treatment for Pteroprocessus obscurinervis

## Additional information

### Artificial Intelligence (AI) use

The authors accept full responsibility for the content of the manuscript, including the disclosure of any use of AI.

Regarding the use of AI in the preparation of this manuscript, the authors declare the following: GPT

Used for: Language, style and writing

ChatGPT was used solely for language editing, including improving the English grammar and syntax of this manuscript. The authors are fully responsible for the content of the manuscript.

### Author ORCIDs

B.P. Schlitt https://orcid.org/0009-0009-7379-0705

### Data availability

All of the data that support the findings of this study are available in the main text.
